# 

**Published:** 1885-05-20

**Authors:** 


### TABLE OF CONTENTS.

ORIGINAL AND SELECTED ARTICLES:

Treatment of a Case of Cerebro-Spinal Meningitis, by J. McF. Gaston, M.D., Professor of Principles and Practice of Surgery in the SOUTHERN MEDICAL COLLEGE, Atlanta, Ga., 161. Pruritus in Diabetes and Icterus, 167. Chronic Catarrh, 169. Alcoholic Hyperæsthesia and Paræsthesia of the Feet, 174 Hepatic Disorders, from a Brief Treatise on Therapeutics, 176. The Intra-Vascular Injection of Blood and other Fluids, 179.

ABSTRACTS AND GLEANINGS:

The Use and Abuse of the Tampon in Abortion, 182. Anæsthetic Agents—The State of the Case, 184. Sodium Salicylate in Neuralgic Headache, 186. Hypophosphite of Lime in Cancer; Precautions to be Adopted in the Treatment of Phthisis, 187. Action of Iodoform in Diabetes: Treatment of Earache; Fluoride of Quinine for Enlarged Spleen: Antidote to Strychnine, 188 Treatment of Bromidrosis of the Feet; Messrs. Wm R. Warner & Co's Concentrated Pepsin; Antipyrin; Extract of Piscidia, 189. Chloride of Methyl Spray as an Anodyne; Valoid of Coca; Evil Effect of Tobacco, 190.

SCIENTIFIC ITEMS:

Petroleum in Russia; Feat of the Divining Rod, 191. The Painless Extinction of Life of Animals; At the Top of Mount Washington; Baron von Schoeler, 192.

PRACTICAL NOTES AND FORMULÆ.

An Inhalation for Coryza; For Ringworm; For Gonorrhœa; Paper for Taking Out Ink Stains; Ginger Ale Easily Made, 193. White's Cough Syrup; Lusk's Aperient Pills; Lotion for Dandruff; Dr. Sabonbiene's Benzoated Ptisan; Coryza; Remedy against Distressing and Fatiguing Coughs, 194.

EDITORIALS AND MISCELLANEOUS.

Editorial Notices; The Transactions of the Medical Association of Georgia, 195. American Medical Association, 196. Book Notices, 198. Receipted; Special Notices, 200.

